# Molecular characterization and clinical outcomes in *EGFR*-mutant *de novo* MET-overexpressed advanced non-small-cell lung cancer

**DOI:** 10.1016/j.esmoop.2021.100347

**Published:** 2021-12-23

**Authors:** J. Mi, Z. Huang, R. Zhang, L. Zeng, Q. Xu, H. Yang, A. Lizaso, F. Tong, X. Dong, N. Yang, Y. Zhang

**Affiliations:** 1 Department of Medical Oncology, Lung Cancer and Gastrointestinal Unit, Hunan Cancer Hospital/The Affiliated Cancer Hospital of Xiangya School of Medicine, Central South University, Changsha, China; 2 Graduate School, University of South China, Hengyang, Hunan, China; 3 Cancer Center, Union Hospital, Tongji Medical College, Huazhong University of Science and Technology, Wuhan, China; 4 Department of Medical Oncology, Qinghai Provincial People's Hospital, Xining, China; 5 Burning Rock Biotech, Guangzhou, China

**Keywords:** *EGFR* mutation, *de novo MET* overexpression/amplification, *EGFR*-*TKI*, crizotinib, non-small-cell lung cancer

## Abstract

**Background:**

Approximately 2%-8% of non-small-cell lung cancer (NSCLC) harbors concurrent epidermal growth factor receptor (*EGFR*) sensitizing mutation and mesenchymal–epithelial transition factor (*MET*) amplification prior to EGFR-tyrosine kinase inhibitor (EGFR-TKI) therapy. This study aimed to investigate the optimal first-line therapeutic options for patients with concurrent *EGFR*-mutant, *MET*-overexpressed/amplified advanced NSCLC.

**Methods:**

A total of 104 treatment-naïve patients with *EGFR*-mutant *de novo* MET-overexpressed advanced NSCLC were identified using immunohistochemistry and stratified to four groups according to treatment regimen: EGFR-TKI monotherapy (*n* = 48), EGFR-TKI combined with either crizotinib (*n* = 9) or chemotherapy (*n* = 12), and chemotherapy (*n* = 35). A subpopulation of 28 patients was also tested with next-generation sequencing (NGS). Objective response rate (ORR) and progression-free survival (PFS) outcomes were analyzed according to treatment strategies and molecular features.

**Results:**

All the patients (*n* = 104) achieved ORR of 36.5% and median PFS (mPFS) of 7.0 months. Baseline clinicopathologic characteristics were similar among the four treatment groups. Compared with chemotherapy, EGFR-TKI monotherapy or EGFR-TKI combination therapy achieved significantly higher ORR (*P* < 0.001) and longer mPFS (*P* = 0.003). No ORR or PFS difference was observed between EGFR-TKI monotherapy and combination therapy. In the NGS-identified population (*n* = 28), patients who received EGFR-TKI plus crizotinib (*n* = 9) achieved similar ORR (88.9% versus 57.9%, *P* = 0.195) and mPFS (9.0 versus 8.5 months, hazard ratio 1.10, 95% confidence interval 0.43-2.55, *P* = 0.45) than those who received EGFR-TKI monotherapy (*n* = 19), regardless of *MET* copy number status. Grade 3/4 rashes were significantly more among patients who received EGFR-TKI plus crizotinib (*P* = 0.026).

**Conclusions:**

Our findings provided clinical evidence that patients with concurrent *EGFR* sensitizing mutation and *de novo MET* amplification/overexpression could benefit from first-line EGFR-TKI monotherapy.

## Highlights

• Concomitant *EGFR* sensitizing mutation and MET overexpression/amplification were detected in 2.6% of lung cancer patients.
• EGFR-TKI monotherapy elicited a higher response rate and longer PFS than chemotherapy.
• EGFR-TKI with or without crizotinib elicited comparable PFS regardless of MET copy number.
• EGFR-TKI monotherapy achieved lower number of grade 3/4 adverse events than EGFR-TKI plus crizotinib.

## Introduction

The prognosis of patients with advanced non-small-cell lung cancer (NSCLC) harboring epidermal growth factor receptor (*EGFR*) sensitizing mutations has significantly improved with *EGFR*-tyrosine kinase inhibitor (TKI) therapy.^1^ However, resistance usually occurs through the activation of EGFR-dependent and EGFR-independent mechanisms. Genomic alterations in *MET*, particularly gene amplification, is one of the most common EGFR-independent mechanisms of acquired resistance to EGFR-TKI.^1^, ^2^, ^3^, ^4^ Mesenchymal–epithelial transition factor (MET) is a receptor tyrosine kinase activated by the binding of hepatocyte growth factor, resulting in the activation of downstream signaling pathways that regulate key cellular functions including cell proliferation, motility, migration, and invasion.^5^ Given its critical role in normal cellular function, aberrations in MET signaling are considered as one of the oncogenic drivers in the development and progression of lung cancer.^6^, ^7^, ^8^ Aberrations in *MET*, either amplification or overexpression, have been reported in 2%-8% of *EGFR*-mutant NSCLCs with no prior exposure to EGFR-TKI therapy.^6^, ^7^, ^8^ Preclinical evidence had demonstrated that the coexistence of *EGFR* mutation and *MET* amplification/overexpression in the same tumor reduces sensitivity to EGFR-TKI, which poses challenges to clinical therapy.^9^ *MET* amplification mediates resistance to EGFR-TKI by activating ERBB3 signaling to activate phosphatidylinositol 3-kinase, thus providing a bypass mechanism of tumor growth.^2^ A potentially effective strategy to overcome this resistance is the combined inhibition of EGFR and MET with targeted agents. Numerous prospective and retrospective clinical studies have demonstrated the efficacy of a combined regimen of EGFR-TKI and MET-TKI in patients who acquired *MET* amplification/overexpression during EGFR-TKI therapy.^10^, ^11^, ^12^, ^13^, ^14^, ^15^, ^16^, ^17^, ^18^, ^19^ However, the optimal first-line treatment strategy for patients with *EGFR*-mutant and *MET*-amplified/overexpressed advanced NSCLC remains controversial. Hence, we conducted a retrospective study to investigate the optimal first-line therapeutic options for patients with dual-driver mutations.

## Patients and methods

### Patient inclusion

A total of 4112 treatment-naïve consecutive patients diagnosed with NSCLC were screened for *EGFR* sensitizing mutation with concurrent *de novo MET* amplification/overexpression. MET overexpression status was assayed using immunohistochemistry (IHC), with a subpopulation of patients also submitted samples for next-generation sequencing (NGS). The samples were collected between September 2015 and January 2021. The patients confirmed to harbor *EGFR* sensitizing mutation concurrent with MET overexpression/amplification were administered with first-line regimen of EGFR-TKI monotherapy, EGFR-TKI combined with crizotinib, EGFR-TKI combined with chemotherapy, or chemotherapy according to the physician's decision and the patient's financial capacity. This project has been reviewed and approved by the Hunan Cancer Hospital Institutional Ethics Committee (2017YYQ-SSB-274). The main inclusion criteria for patients were as follows: age >18 years, stage IIIB to IV locally advanced nonresectable disease or advanced disease according to the 8th American Joint Committee on Cancer Staging System, histologically confirmed lung adenocarcinoma/squamous cell carcinoma, concurrent *EGFR* sensitizing mutations and *MET* amplification/overexpression detected by NGS or IHC at baseline, and no prior systemic treatment of chemotherapy or targeted agents.

### Immunohistochemistry

The patient's tumor slides were prepared from tissue biopsy samples fixed in 10% buffered formalin solution and embedded in paraffin. The antigens were probed with MET (D1C1 antibody) and detected with peroxidase-conjugated secondary antibody. MET positivity was defined as an above-median histochemistry score (H-score) and by staining intensity of +2 or +3 in >50% of tumor cells. MET expression was independently evaluated by two pathologists.

### NGS

Patient samples were submitted for NGS-based analysis to Burning Rock Biotech, a College of American Pathologists-accredited, Clinical Laboratory Improvement Amendments-certified clinical laboratory. In brief, a minimum of 50 ng of DNA isolated from the tissue biopsy or blood samples obtained from the patients was processed accordingly for NGS using commercially available panels targeting 168 cancer-related genes and sequenced on a NextSeq 500 (Illumina, San Diego, CA) with paired-end reads with a target sequencing depth of 1000× for tissue samples and 100 00× for plasma samples using optimized protocols (Burning Rock Biotech, Guangzhou, China).^20^ The capture panel interrogated whole exons and critical introns for the eight classic NSCLC oncogenic drivers, which include *EGFR*, *ALK*, *BRAF*, *ERBB2*, *KRAS*, *MET*, *RET*, and *ROS1*. The sequencing analyses were performed using optimized bioinformatics pipeline for somatic variant calling that involved accurate identification of base substitutions, small insertions–deletions, copy number (CN) variations, and genomic rearrangements as described previously.^20^ *MET* amplification was defined as mean gene CN, with high CN defined as a cut-off of ≥5.

### Assessment of treatment outcome

All patients underwent radiological response evaluation every 4 weeks from the start of the treatment regimen until treatment discontinuation due to toxicity or radiologically confirmed disease progression. Treatment response was evaluated according to RECIST version 1.1. Objective response rate (ORR) was defined as the proportion of patients who achieved complete response (CR) and partial response (PR). Disease control rate (DCR) was defined as the proportion of patients who achieved CR, PR, and stable disease (SD). Adverse events were evaluated based on the Common Terminology Criteria for Adverse Events version 4.0.

### Statistical analysis

All statistical analyses were performed as two-sided tests using SPSS software (version 22 or GraphPad Prism (version 8.3.0). Chi-square test was performed to compare differences between groups. Kaplan–Meier with log-rank statistics was performed to determine the median survival. Cox proportional hazards model was used for multivariate survival analysis. Hazard ratios (HRs) were estimated using the Schoenfeld residuals, with corresponding 95% confidence intervals (CIs). Statistical significance was defined as *P* < 0.05.

## Results

### Patient characteristics

**Figure 1:**
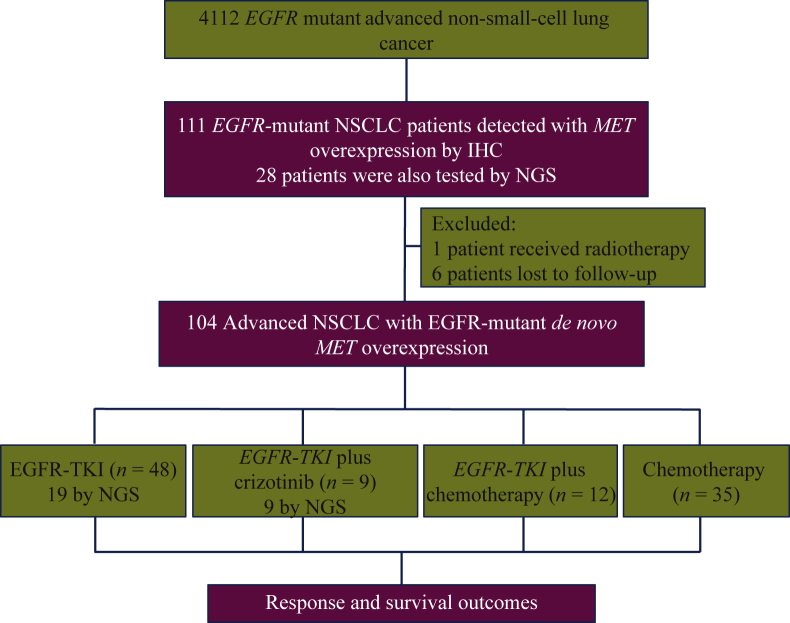
Flow diagram of the study design. EGFR, epidermal growth factor receptor; MET, mesenchymal–epithelial transition factor; TKI, tyrosine kinase inhibitor.

Figure 1 summarizes our study design. Of the 4112 treatment-naïve patients screened, concurrent *EGFR* sensitizing mutation and MET overexpression were detected in 2.7% (111/4112) of patients. A total of 104 treatment-naïve NSCLC patients with concomitant *EGFR* mutation and MET overexpression/amplification at baseline were included in the study. The median age of patients at diagnosis was 56 years (range 27-84 years). The cohort comprised 103 (99%) patients with histological diagnosis of adenocarcinoma, 92 (88.5%) with stage IV disease at diagnosis, and 72 (69.2%) were never smokers (Table 1).

**Table 1:** Patient characteristics

	Total (*n* = 104)	*P*	*EGFR* mutation with *de novo MET* amplification (*n* = 104)				*P*
			EGFR-TKI^a^ monotherapy (*n* = 48)	EGFR-TKI plus crizotinib (*n* = 9)	EGFR-TKI plus chemotherapy (*n* = 12)	Chemotherapy (*n* = 35)	
Age, median (range), years	56 (27-84)		56 (31-84)	52 (27-66)	56 (47-75)	57 (44-73)	0.728
Sex		0.907					0.501
Male, *n* (%)	52 (50.0)		24 (50)	6 (66.7)	4 (33.3)	18 (51.4)	
Female, *n* (%)	52 (50.0)		24 (50)	3 (33.3)	8 (66.7)	17 (48.6)	
Smoking history		0.723					0.544
Never smoker (no history of smoking), *n* (%)	72 (69.2)		16 (33.3)	5 (55.6)	2 (16.7)	10 (28.6)	
Former smoker (previous history of smoking), *n* (%)	32 (30.8)		32 (66.7)	4 (44.4)	10 (83.3)	25 (71.4)	
Histology		0.894					0.758
Adenocarcinoma, *n* (%)	103 (99.0)		47 (97.9)	9 (100.0)	12 (100.0)	35 (100.0)	
Squamous cell carcinoma, *n* (%)	1 (1.0)		1 (2.1)	0 (0.0)	0 (0.0)	0 (0.0)	
Stage		0.204					0.511
Stage IIIb, *n* (%)	12 (11.5)		5 (10.4)	1 (11.1)	0 (0.0)	6 (17.1)	
Stage IV, *n* (%)	92 (88.5)		43(89.6)	8 (88.9)	12 (100.0)	29 (82.9)	
Brain metastasis at baseline		0.280					0.479
Yes, *n* (%)	31 (29.8)		37 (77.1)	7 (77.8)	7 (58.3)	23 (65.7)	
No, *n* (%)	73 (70.2)		11 (22.9)	2 (22.2)	5 (41.7)	12 (34.3)	
EGFR mutation status		0.006					0.420
Exon 19 deletion, *n* (%)	65 (62.5)		34 (70.8)	6 (66.7)	8 (66.7)	17 (48.6)	
Exon 21 L858R, *n* (%)	26 (25.0)		10 (20.8)	1 (11.1)	3 (25.0)	12 (34.3)	
Uncommon EGFR mutation, *n* (%)	13 (12.5)		4 (8.4)	2 (22.2)	1 (8.3)	6 (17.1)	

EGFR, epidermal growth factor receptor; MET, mesenchymal–epithelial transition factor; TKI, tyrosine kinase inhibitor.

a EGFR-TKI refers to gefitinib, erlotinib, afatinib, or osimertinib.

### Baseline molecular profiling

The 104 patients had MET overexpression detected by IHC, with 89 (85.7%) patients having high MET overexpression (IHC 3+) and 15 (14.3%) patients having low MET overexpression (IHC 2+). Of all the patients, 65 (62.5%) harbored *EGFR* exon 19 deletion (19del), 26 (25.0%) had *EGFR* exon 21 L858R, and 13 (12.5%) had uncommon *EGFR* mutations, including *EGFR* exon 18 L861Q, G719X (Supplementary Figure S1, available at https://doi.org/10.1016/j.esmoop.2021.100347). Of the 104 patients, 28 also submitted samples for NGS analysis. Among them, concurrent mutations in oncogenic driver genes [*KRAS* (*n* = 2), *BRAF* (*n* = 3), *EGFR* amplification (*n* = 11), and/or *ERBB2* (*n* = 2)] were also detected in 16 (57.1%) patients, while 5 (17.9%) patients were detected with concurrent mutations in tumor suppressor genes (*TP53*, *RB1*; Supplementary Figure S2, available at https://doi.org/10.1016/j.esmoop.2021.100347). *TP53* mutation was detected in six patients (25%). No patient was detected with *MET* exon 14 skipping mutations. There was no difference in baseline molecular profiles between patients who received EGFR-TKI monotherapy and EGFR-TKI plus crizotinib.

### Treatment and outcomes

**Figure 2:**
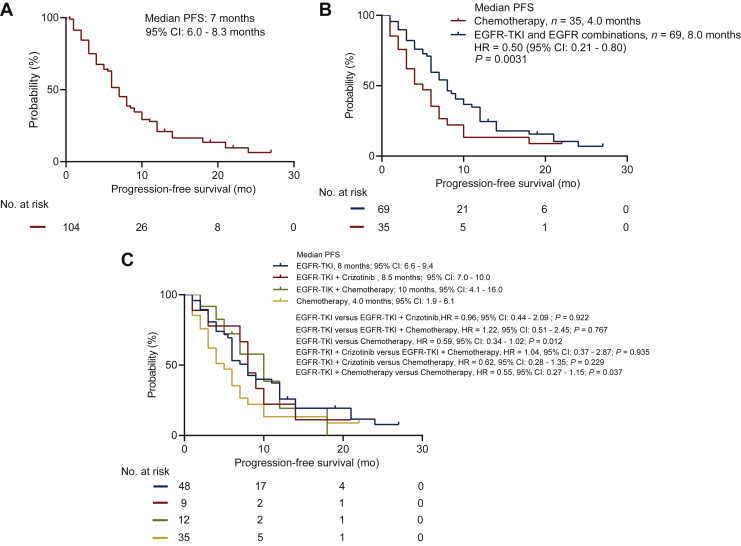
(A) The progression-free survival curve of all patients of our cohort. (B) The progression-free survival curve of patients who received any EGFR-TKI-based therapy (either monotherapy or combination therapy; *n* = 69) versus chemotherapy (*n* = 35). (C) The progression-free survival curve of four groups: EGFR-TKI monotherapy (*n* = 48), EGFR-TKI combined with chemotherapy (*n* = 12), EGFR-TKI combined with crizotinib (*n* = 9), and chemotherapy (*n* = 35). EGFR, epidermal growth factor receptor; TKI, tyrosine kinase inhibitor.

The overall median progression-free survival (mPFS) of the cohort was 7.0 months (95% CI 6.0-8.3; Figure 2A). The clinical outcomes are presented in Supplementary Table S1, available at https://doi.org/10.1016/j.esmoop.2021.100347. Of all patients, 48 (46.2%) received first-line EGFR-TKI monotherapy, 9 (8.7%) received crizotinib combined with EGFR-TKI, 12 (11.5%) received EGFR-TKI combined with chemotherapy, and 35 (33.6%) received chemotherapy. Baseline clinical characteristics were similar among the groups, including rate of baseline brain metastasis (Table 1).

Of the 48 patients who received EGFR-TKI monotherapy, 22 achieved PR, and 19 had SD after two cycles of treatment, achieving an ORR of 45.8% and DCR of 85.4%. Seven (14.6%) patients failed to benefit from EGFR-TKI monotherapy. The nine patients in the EGFR-TKIs plus crizotinib combination therapy group achieved an ORR and DCR of 88.9%. The EGFR-TKIs plus chemotherapy group reached an ORR of 41.7% and DCR of 100%. Of the 35 patients who received chemotherapy, 3 patients achieved PR and 27 had SD after two cycles of treatment, achieving an ORR of 8.6% and DCR of 85.7%. Patients who received EGFR-TKI-based regimens, including EGFR-TKI monotherapy, or combined with either crizotinib or chemotherapy had significantly better ORR than those who received chemotherapy (45.8%, 88.9%, 41.7% versus 8.6%, *P <* 0.001). No statistical difference in ORR was found among the patients who received EGFR-TKI monotherapy or combined with crizotinib or chemotherapy (*P* = 0.993, Supplementary Table S1, available at https://doi.org/10.1016/j.esmoop.2021.100347).

The patients who received EGFR-TKI monotherapy or EGFR-TKI combined with either crizotinib or chemotherapy had significantly longer PFS than those who received chemotherapy (mPFS: 8.0 months versus 4.0 months, *P* = 0.031, Figure 2B). Patients who received EGFR-TKI monotherapy or EGFR-TKI combined with chemotherapy achieved a significantly better mPFS outcome as compared with chemotherapy (8.0 months versus 4.0 months, *P* = 0.012, and 10.0 months versus 4.0 months, *P* = 0.037; Figure 2C). There was no significant PFS difference between the EGFR-TKI combined with crizotinib group and the EGFR-TKI monotherapy group (*P* = 0.922), the EGFR-TKI combined with chemotherapy group (*P* = 0.935), and the chemotherapy group (*P* = 0.229; Figure 2C).

**Figure 3:**
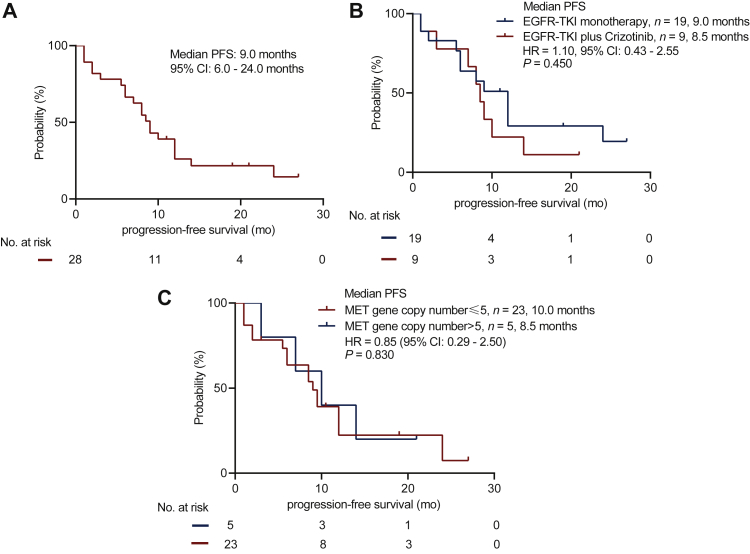
(A) The progression-free survival curve of all patients whose MET status was evaluated by both NGS and IHC. (B) The progression-free survival curve of patients detected by NGS and IHC who received EGFR-TKI monotherapy (*n* = 19) or EGFR-TKI combined with crizotinib (*n* = 9). (C) The progression-free survival curve of patients with MET gene copy number ≥5 or <5. EGFR, epidermal growth factor receptor; IHC, immunohistochemistry; MET, mesenchymal–epithelial transition factor; NGS, next-generation sequencing; TKI, tyrosine kinase inhibitor.

We analyzed the clinical outcomes of the patients with *MET* amplification detected by NGS. All 28 patients achieved an mPFS of 9.0 months (Figure 3A). Among them, 19 patients were treated with EGFR-TKI monotherapy and 9 patients received EGFR-TKI combined with crizotinib. Both groups had similar concomitant mutations at baseline (Supplementary Table S2, available at https://doi.org/10.1016/j.esmoop.2021.100347) and achieved similar PFS (9.0 months versus 8.5 months, *P* = 0.45; Figure 3B and Supplementary Figures S3 and S4, available at https://doi.org/10.1016/j.esmoop.2021.100347). Moreover, both groups achieved similar ORR (57.9% versus 88.9%, *P* = 0.153) and DCR (84.2% versus 100%, *P* = 0.530, Supplementary Table S3, available at https://doi.org/10.1016/j.esmoop.2021.100347). Five patients with high CN *MET* amplification (NGS-CN ≥5) achieved PR after two cycles of treatment, with an ORR and DCR of 100%, and 23 patients with low CN *MET* amplification (NGS-CN <5) had an ORR of 60.9% and DCR of 82.6%, which resulted in no statistical difference (Supplementary Table S4, available at https://doi.org/10.1016/j.esmoop.2021.100347). Similarly, there was no statistical difference in PFS between CN ≥5 group and CN <5 group (10.0 months versus 8.5 months, *P* = 0.830; Figure 3C). Only one patient was detected with MET CN of >10.

### Adverse events

Table 2 summarizes the adverse events reported by the 9 patients treated with EGFR-TKI and crizotinib combination therapy and 19 patients who received EGFR-TKI monotherapy. Grade 1/2 skin rashes were the most frequent adverse events, which occurred in 33.3% of the patients who received EGFR-TKI monotherapy and 22.2% of the patients who received EGFR-TKI and crizotinib combination therapy. No unexpected grade 3/4 event was observed in patients who received EGFR-TKI monotherapy or combination therapy. Major grade 3/4 adverse events in those who received combination therapy included diarrhea (*n* = 1), elevated aspartate aminotransferase (*n* = 1), and nausea/vomiting (*n* = 1). No difference was observed in grade 1/2 adverse events between the EGFR-TKI monotherapy group and the EGFR-TKI plus crizotinib group; however, grade 3/4 rashes were significantly higher in the EGFR-TKI plus crizotinib group (33.3% versus 0%, *P* = 0.026, Table 2), which were managed via dose reduction. No patient discontinued the treatment regimen due to treatment-related toxicities.

**Table 2:** Adverse events

	Grade 1/2 (*n*, %)		*P*	Grade 3/4 (*n*, %)		*P*
	EGFR-TKI monotherapy (*n* = 19)	EGFR-TKI+ crizotinib (*n* = 9)		EGFR-TKI monotherapy (*n* = 19)	EGFR-TKI+ crizotinib (*n* = 9)	
Rash	6 (31.5)	2 (22.2)	0.103	0 (0)	3 (33.3)	0.026
Diarrhea	5 (26.3)	1 (11.1)	0.630	0 (0)	1 (11.1)	0.321
Elevated aspartate aminotransferase	4 (21)	1 (11.1)	1.000	1 (5.3)	1 (11.1)	1.000
Nausea/vomiting	2 (10.5)	2 (22.2)	0.574	0 (0)	1 (11.1)	0.321
Neutropenia	1 (5.7)	0 (0)	1.000	0 (0)	0 (0)	—
Vision impairment	0 (0)	1 (11.1)	0.321	0 (0)	0 (0)	—

EGFR, epidermal growth factor receptor; TKI, tyrosine kinase inhibitor.

## Discussion

Alterations in MET resulting from either gene amplification or exon 14 skipping have been considered as therapeutic targets in NSCLC and clinically benefit from treatment with crizotinib or other MET-TKIs.^21^, ^22^, ^23^ However, a subset of treatment-naïve NSCLCs would present with concurrent mutations in *EGFR* and *MET*,^6^ which has been implicated in the primary resistance to EGFR-TKI in NSCLC.^2^ The combination of EGFR-TKI and crizotinib was shown to be more effective than EGFR-TKI monotherapy in response to *MET* amplification/overexpression-mediated EGR-TKI resistance.^10^, ^11^, ^12^, ^13^, ^14^, ^15^, ^16^ Wang et al.^24^ reported that MET FISH positivity (HR 2.83, 95% CI 1.37-5.86) was an independent predictor for poorer PFS in patients with *EGFR*-mutant NSCLC who received first-line EGFR-TKI treatment after adjustment for multiple factors including BIM, ALK, KRAS, PIK3CA, PTEN, MET, etc. Studies on EGFR-TKI-naïve patient-derived xenograft model harboring concurrent *EGFR* L858R and *MET* amplification demonstrated partial sensitivity with EGFR-TKI monotherapy but achieved CR with EGFR-TKI combined with crizotinib, suggesting that both *EGFR* and *MET* are oncogenic drivers that are sensitive to inhibition.^9^^,^^14^, ^15^, ^16^ Furthermore, a case report demonstrated the effectiveness of erlotinib plus crizotinib in a patient with lung adenocarcinoma harboring concurrent *de novo EGFR* L858R and *MET* amplification (FISH MET/centromere of chromosome 7 ratio >15).^11^ This preclinical and clinical evidence implicates *MET* amplification in mediating primary resistance to EGFR-TKI. However, clinical evidence has shown that EGFR-TKI monotherapy is still effective for patients harboring concurrent actionable mutations in *EGFR* and *MET*.^3^ The question remains whether MET overexpression/amplification detected at baseline is actionable or not. Because of the lack of consensus on the optimal therapy for this subset of patients, the clinical management of these patients in the real world is more varied, with some practitioners administering the standard of care EGFR-TKI monotherapy, while others choose alternative treatment strategies including EGFR-TKI combined with either MET-TKI or chemotherapy or chemotherapy regimen alone. Therefore, it is necessary to explore the clinical outcomes of patients harboring rare double driver mutations to understand which of these therapeutic strategies could provide the best clinical benefit. To the best of our knowledge, our retrospective cohort study is the first to investigate the efficacy of EGFR-TKI alone or in combination with either crizotinib or chemotherapy in treatment-naïve NSCLC harboring concurrent *EGFR* sensitizing mutation and MET overexpression.

In our cohort, the proportion of treatment-naïve patients with advanced NSCLC detected with *EGFR* mutations and MET overexpression at baseline was 2.7%, which is consistent with previous reports.^6^ The mPFS of the patients with dual drivers who received EGFR-TKI monotherapy was ∼8.0 months, which was shorter than the data in previous clinical trials for first-line EGFR-TKI therapy of patients with *EGFR*-mutant NSCLC.^25^, ^26^, ^27^ Similarly, the mPFS was 4 months for patients with dual drivers who received chemotherapy, which was also inferior to the data in previous studies.^25^, ^26^, ^27^ These data suggest that harboring concurrent *EGFR* mutation and MET overexpression/amplification at baseline has a negative impact on treatment outcome. However, we did not observe significantly better clinical outcomes for patients who received EGFR-TKI in combination with crizotinib. For patients with NGS-based *MET* CN data, no significant difference in either ORR or PFS was observed, regardless of CN ≥5 or CN <5, while these results are consistent with previous data.^3^ A male patient with stage IV lung adenocarcinoma who had NGS-detected *MET* amplification CN of 12 was treated with EGFR-TKI combined with crizotinib and achieved PR lasting for 14 months. This might suggest that the combination therapy could benefit patients with higher *MET* CN; however, more evidence is needed to validate this observation. Moreover, in patients who had both NGS and IHC-based MET alteration data, EGFR-TKI monotherapy (*n* = 19) or EGFR-TKI combined with crizotinib (*n* = 9) had a similar PFS (9.0 months versus 8.5 months, *P* = 0.45), ORR (57.9% versus 88.9%, *P* = 0.153), and DCR (84.2% versus 100%, *P* = 0.530). The co-occurrence of both *EGFR* mutation and *MET* amplification, particularly at baseline, indicates a more complex genetic heterogeneity of the tumor in these patients. Despite high MET overexpression or *MET* amplification, respectively evaluated using IHC or NGS, we did not observe a synergistic antitumor effect with the coadministration of crizotinib and EGFR-TKI. Based on the similar clinical benefit observed for EGFR-TKI with or without crizotinib, it was evident that the aberrant EGFR pathway is a strong oncogenic driver, which was sensitive to inhibition with EGFR-TKI, while the aberrant MET pathway was oncogenic as shown by the rapid disease progression, but was not highly sensitive to crizotinib inhibition to the point that its antitumor activity did not result in better survival outcomes. We speculate that this is due to the multiple kinase activities of crizotinib that make it less selective; it is also possible that other more selective MET-TKI, such as tepotinib, capmatinib, or savolitinib, could be more efficacious in this scenario and requires more clinical studies. The combination of EGFR-TKI and selective MET-TKI had shown promising clinical efficacy and safety in the early phase of clinical trials.^17^, ^18^, ^19^ MET positivity for inclusion of patients in these clinical trials was determined by either FISH alone (MET/centromere of chromosome 7 ratio of ≥2 or MET gene CN ≥5),^19^ the combination of IHC (MET 2+/3+) and FISH,^17^ or the combination of IHC (MET 3+ expression in ≥50% of tumor cells), FISH, and NGS (≥5 copies of MET).^18^ However, these clinical trials investigated the efficacy and safety of these combinations in smaller cohort of patients who progressed from prior EGFR-TKI therapy. It remains to be determined whether these combination regimens would be equally effective and safe for treatment-naïve patients and in a larger cohort. A clinical trial that investigated erlotinib combined with onartuzumab, an antibody against MET, has shown promising results in preclinical and phase II clinical studies but failed to demonstrate significant results in the phase III trials that compared erlotinib with or without onartuzumab in previously treated patients with *EGFR*-positive MET-overexpressed NSCLC.^28^ Because erlotinib and onartuzumab showed good antitumor activity *in vitro*,^28^ it is possible that MET overexpression assessed by IHC is not an effective biomarker of response to MET-TKIs. Numerous studies have demonstrated that MET overexpression assayed using IHC does not reflect *MET* amplification, while *MET* amplification assayed by either FISH or NGS was more reliable and more correlated with treatment response with MET-TKI.^17^^,^^29^, ^30^, ^31^ However, with the complexity of data interpretation for FISH assays as well as the need for extra tissue samples, we find multigene panel NGS to be a more convenient and suitable genomic assay. Li et al.^32^ demonstrated that NGS-based detection of *MET* amplification with higher CN (>4) had significantly longer PFS with crizotinib monotherapy.^32^ Their subgroup analysis on patients with dual *EGFR* and *MET* amplification (*n* = 11) demonstrated DCR of 72.7% (PR: *n* = 2; SD: *n* = 6; progressive disease: *n* = 3) and had mPFS of 2.8 months with single-agent crizotinib, which were not significantly different from patients with single *MET* amplification. The initial benefit is very short-lived with crizotinib monotherapy, suggesting that patients with dual *EGFR*/*MET* mutations could benefit more from an EGFR-TKI-containing regimen. Further analysis of adverse events between the two groups showed no significant difference in grade 1/2 adverse events; however, grade 3/4 rashes were more frequent in the combination therapy group (*P* = 0.026). These data suggest that the clinical outcomes of patients with dual drivers who received EGFR-TKI monotherapy are not inferior to the combination therapy and had fewer grade 3/4 adverse events, thus suggesting that the EGFR-TKI monotherapy is safer than the combination therapy.

The limitations of our study included its retrospective nature and the inclusion of a small cohort; hence our findings are exploratory and hypothesis-generating. A larger multicenter prospective study is needed to further confirm our findings. A larger cohort could allow more subgroup analysis for treatment outcomes, including different NGS-based *MET* CN values, and the presence of concurrent mutations.

In conclusion, our study provided clinical evidence of the effectiveness of EGFR-TKI or EGFR-TKI combined with crizotinib/chemotherapy in patients with concurrent actionable mutations in *EGFR* and *MET*. Moreover, EGFR-TKI monotherapy achieved similar efficacy and fewer toxicities as compared with EGFR-TKI plus crizotinib.
