# Supplementary material for: Molecular characterization and clinical outcomes in EGFR-mutant de novo MET-overexpressed advanced non-small-cell lung cancer
Source: ESMO Open. 2021 Dec 23;7(1):100347. doi: 10.1016/j.esmoop.2021.100347 (PMC8717426; doi:10.1016/j.esmoop.2021.100347)
Supplement: Supplementary Figures S1-S4 [file mmc1.pptx]

## Slide 1
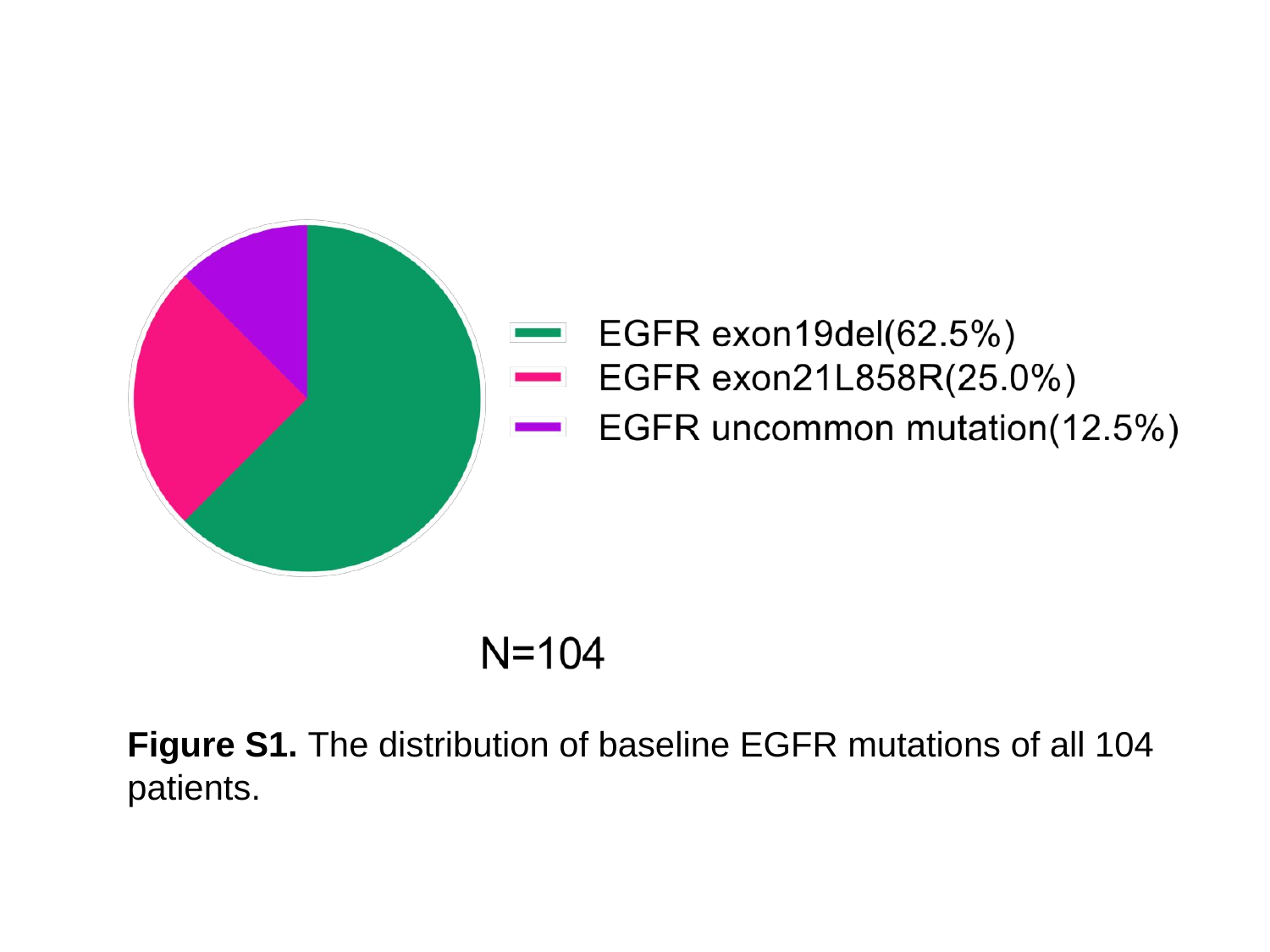

Figure S1. The distribution of baseline EGFR mutations of all 104 patients.

## Slide 2
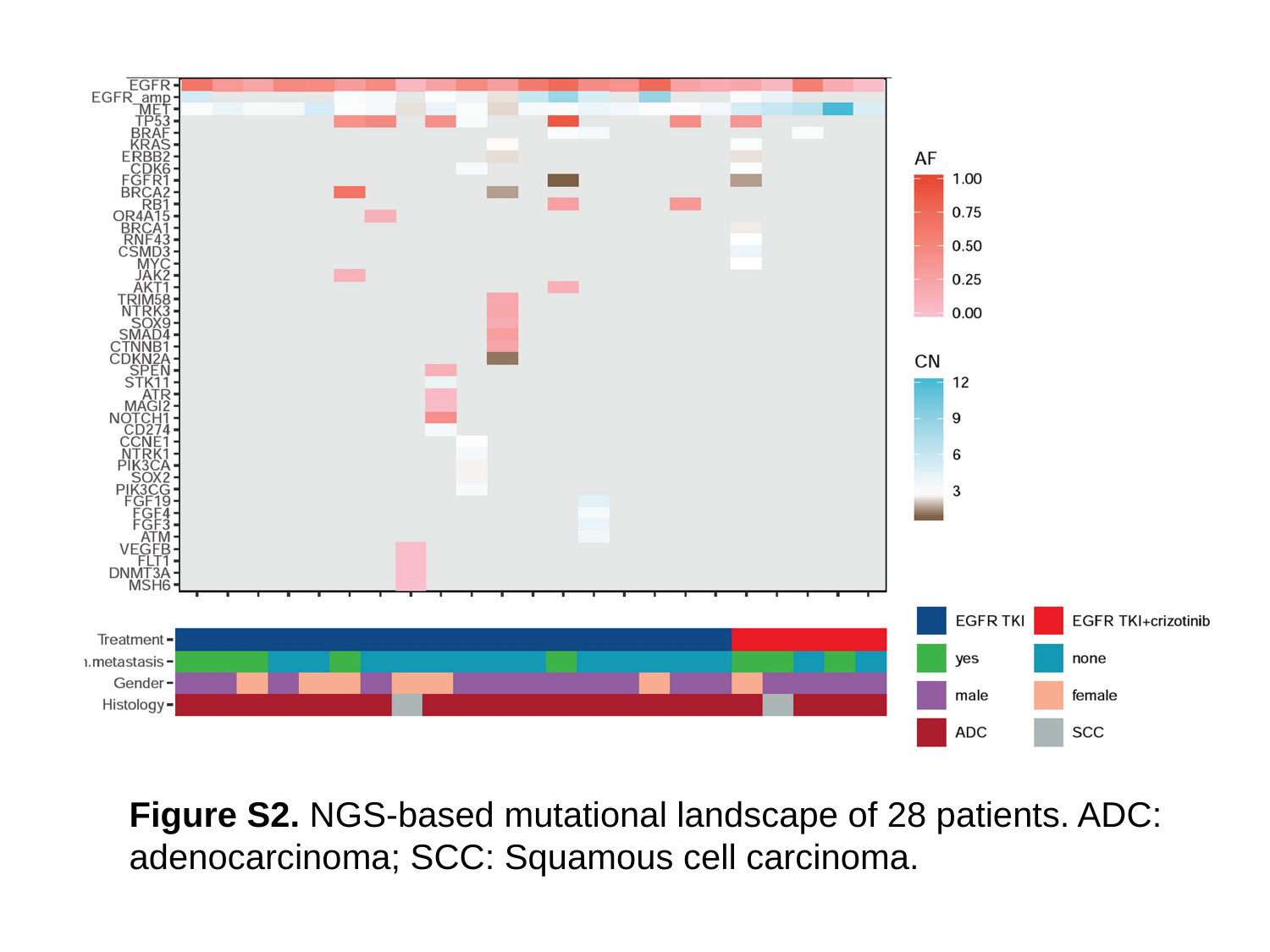

Figure S2. NGS-based mutational landscape of 28 patients. ADC: adenocarcinoma; SCC: Squamous cell carcinoma.

## Slide 3
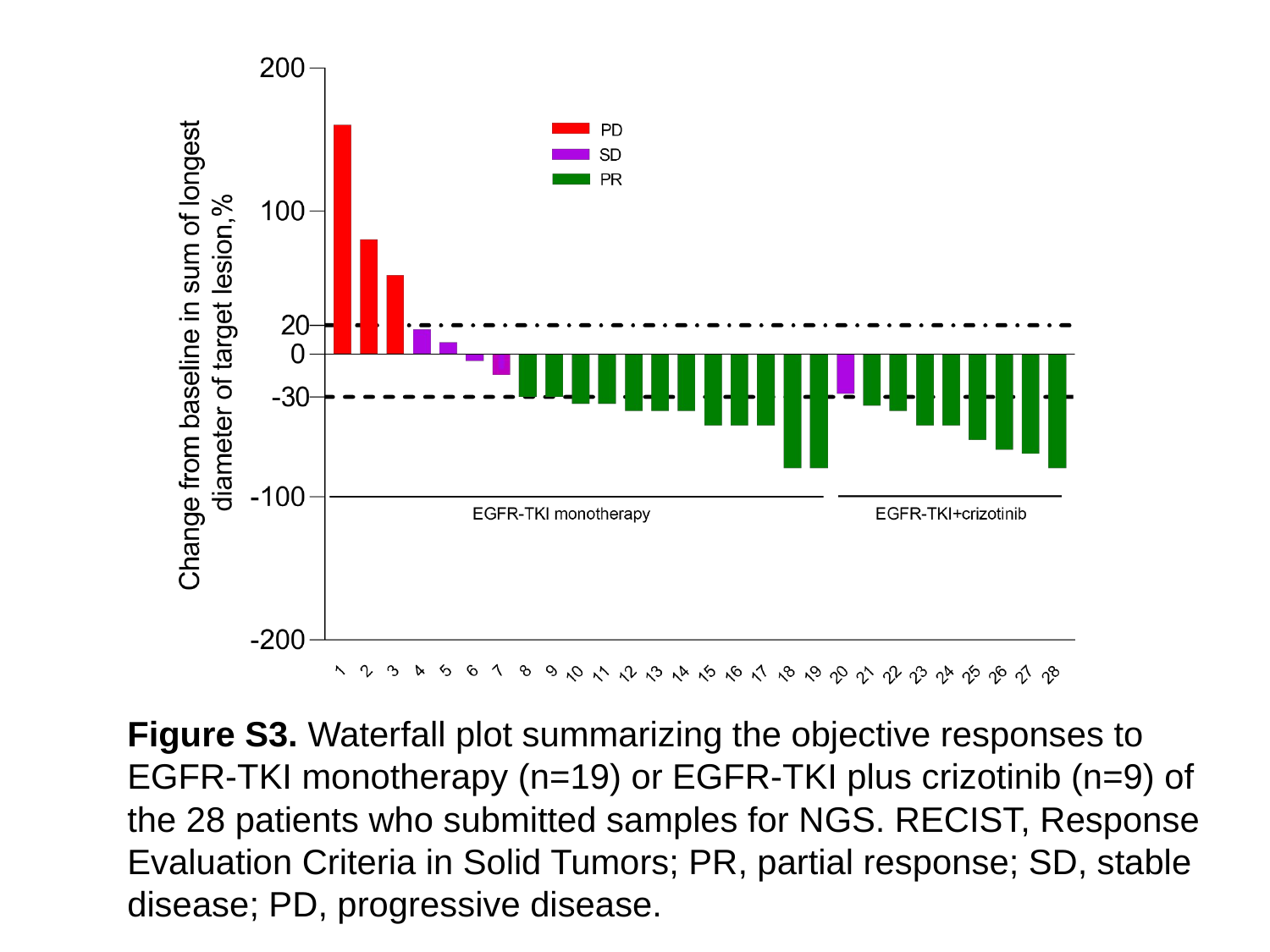

Figure S3. Waterfall plot summarizing the objective responses to EGFR-TKI monotherapy (n=19) or EGFR-TKI plus crizotinib (n=9) of the 28 patients who submitted samples for NGS. RECIST, Response Evaluation Criteria in Solid Tumors; PR, partial response; SD, stable disease; PD, progressive disease.

## Slide 4
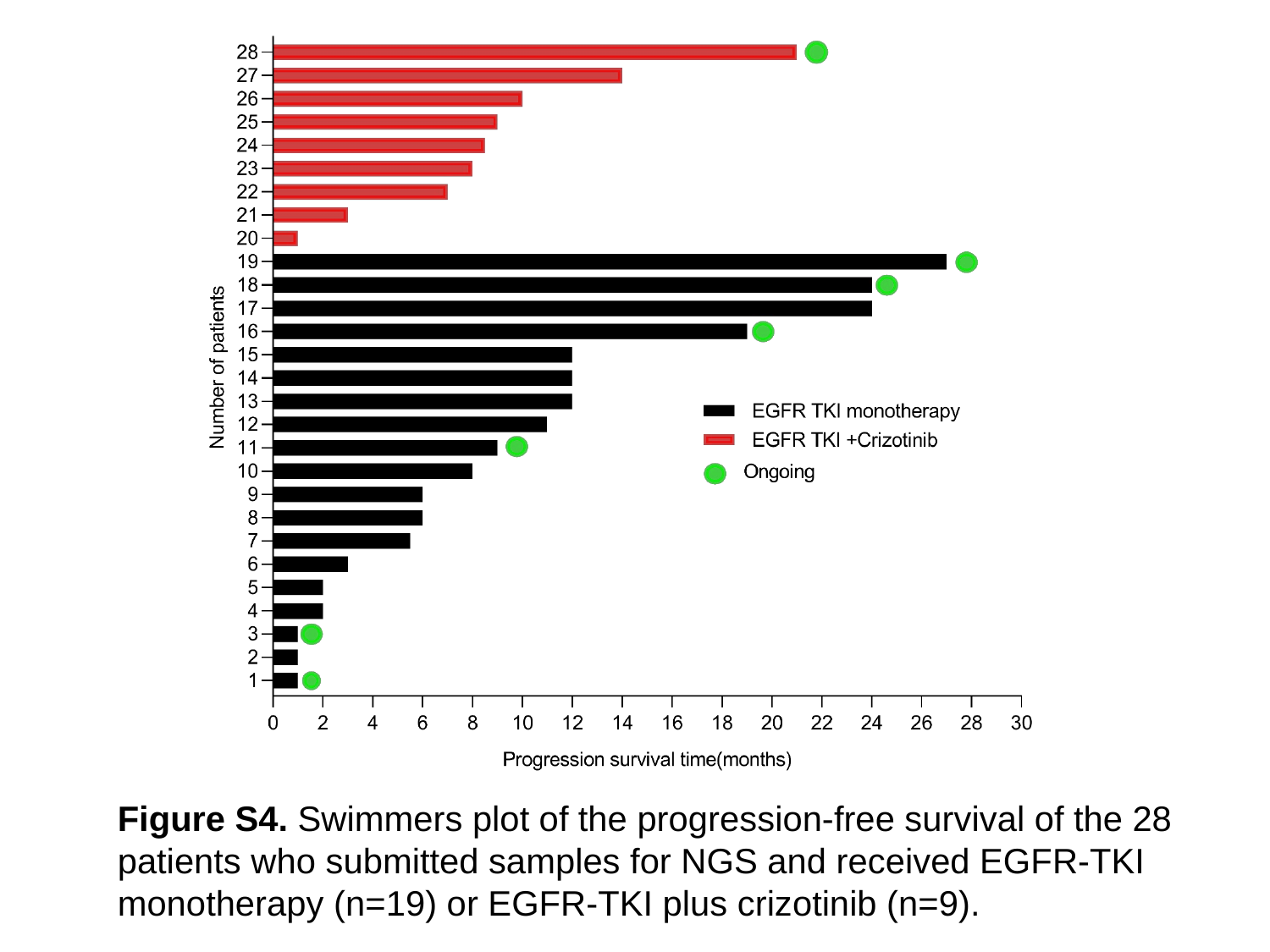

Figure S4. Swimmers plot of the progression-free survival of the 28 patients who submitted samples for NGS and received EGFR-TKI monotherapy (n=19) or EGFR-TKI plus crizotinib (n=9).
