# Supplementary material for: Molecular characterization and clinical outcomes in EGFR-mutant de novo MET-overexpressed advanced non-small-cell lung cancer
Source: ESMO Open. 2021 Dec 23;7(1):100347. doi: 10.1016/j.esmoop.2021.100347 (PMC8717426; doi:10.1016/j.esmoop.2021.100347)
Supplement: Supplementary Tables S1-S4 [file mmc2.docx]

**Table S1.** Treatment outcomes of the cohort

|  | Total (n=104) | EGFR mutation with de novo MET amplification (n=104) | | | | *p* |
| --- | --- | --- | --- | --- | --- | --- |
|  |  | EGFR-TKI monotherapy (n=48) | EGFR-TKI plus crizotinib (n=9) | EGFR-TKI plus chemotherapy (n=12) | Chemotherapy(n=35) |  |
| CR | 0(0%) | 0(0.0%) | 0(0.0%) | 0(0.0%) | 0(0.0%) |  |
| PR | 38(36.5%) | 22(45.8%) | 8(88.9%) | 5(41.7%) | 3(8.6%) |  |
| SD | 53(51.0%) | 19(39.6%) | 1(0.0%) | 7(58.3%) | 27(77.1%) |  |
| PD | 13(12.5%) | 7(14.6%) | 0(11.1%) | 0(0.0%) | 5(14.3%) |  |
| ORR | 38(36.5%) | 22(45.8%) | 8(88.9%) | 5(41.7%) | 3(8.6%) | <0.001 |
| DCR | 91(87.5%) | 41(85.4%) | 9(100%) | 12(100.0%) | 30(85.7%) | 0.693 |

Abbreviations: CR, complete response; DCR, disease control rate; EGFR-TKI, epidermal growth factor receptor tyrosine kinase inhibitor; ORR, objective response rate; PD, progressive disease; PR, partial response; SD, stable disease

**Table S2.** Distribution of the cohort according to concomitant mutations harbored

|  | Total (n=28) | EGFR mutation with de novo MET amplification (n=28) | | |
| --- | --- | --- | --- | --- |
|  |  | EGFR-TKI monotherapy (n=19, 67.9%) | EGFR-TKI plus crizotinib (n=9, 32.1%) | p |
| No other concomitant mutations | 7(25%) | 3(15.8%) | 4(44.4%,) | 0.277 |
| With other driver mutations | 16(57.1%) | 12(63.2%) | 4(44.4%) | 0.828 |
| With tumor suppressor genes | 5(17.9%) | 4(21%) | 1(11.2%) | 1.000 |

**Table S3.** Treatment outcomes of all patients who were positive for NGS-based *MET* amplification

|  | Total (n=28) | EGFR-TKI monotherapy (n=19) | EGFR-TKI plus crizotinib (n=9) | *p* |
| --- | --- | --- | --- | --- |
| CR | 0（0.0%） | 0（0.0%） | 0(0.0%) |  |
| PR | 19(67.9%) | 11(57.9%) | 8(88.9%) |  |
| SD | 5(17.9%) | 5(26.3%) | 1(0.0%) |  |
| PD | 4(14.3%) | 3(15.8%) | 0(11.1%) |  |
| ORR | 19(67.9%) | 11(57.9%) | 8(88.9%) | 0.195 |
| DCR | 24(85.7%) | 16（84.2%） | 9(100%) | 0.530 |

Abbreviations: CR, complete response; DCR, disease control rate; EGFR-TKI, epidermal growth factor receptor tyrosine kinase inhibitor; NGS, next-generation sequencing; ORR, objective response rate; PD, progressive disease; PR, partial response; SD, stable disease

**Table S4.** Treatment outcomes stratified according to NGS-based MET copy number (n=28)

|  | Total (n=28) | NGS +(n=28) | | *p* |
| --- | --- | --- | --- | --- |
|  |  | MET CN＞5 (n=5) | MET CN＜5 (n=23) |  |
| CR | 0(0.0%) | 0(0.0%) | 0(0.0%) |  |
| PR | 19(67.9%) | 5(100.0%) | 14(60.9%) |  |
| SD | 5(17.9%) | 0(0.0%) | 5(21.7%) |  |
| PD | 4(14.3%) | 0(0.0%) | 4(17.4%) |  |
| ORR | 19(67.9%) | 5(100.0%) | 14(60.9%) | 0.144 |
| DCR | 24(85.7%) | 5(100.0%) | 19(82.6%) | 1.000 |

Abbreviations: CN, copy number; CR, complete response; DCR, disease control rate; NGS, next-generation sequencing; ORR, objective response rate; PD, progressive disease; PR, partial response; SD, stable disease
